# Long-Term Survival of Cutaneous Malignant Melanoma with Metastasis to Paranasal Sinuses: A Case Report and Literature Review

**DOI:** 10.22086/gmj.v0i0.860

**Published:** 2018-12-31

**Authors:** Kazem Anvari, Mohammad Reza Majidi, Mahdi Razmara Ferezghi, Bahereh Parkam, Seyed Alireza Javadinia

**Affiliations:** ^1^Cancer Research Center, Faculty of Medicine, Mashhad University of Medical Sciences, Mashhad, Iran; ^2^Ear, Nose, Throat Research Center, Ghaem Hospital, School of Medicine, Mashhad University of Medical Sciences, Mashhad, Iran; ^3^Student Research Committee, Faculty of Medicine, Mashhad University of Medical Sciences, Mashhad, Iran

**Keywords:** Melanoma, Cutaneous Malignant, Paranasal Sinuses, Survival

## Abstract

**Background:**

Malignant melanoma (MM) usually present with metastases to unexpected regions of the body. Metastatic MM is a highly lethal condition, and the median survival in this setting is 6 to 7.5 months; however, few reports rarely describe long-term after chemotherapy.

**Case report:**

We describe a 31-year-old man with MM, which got metastatic (to paranasal sinuses) after local and systemic therapy showed complete responses with long-term survival after endonasal endoscopic metastasectomy and radiotherapy of the nasal cavity, paranasal sinuses, and base of the skull.

**Conclusion:**

Although long-term survival is rare, few reports describe cases after chemotherapy. MM could be associated with metastasis to any regions and clinicians should be aware of its behavior and perform complete investigation in the presence of any suspicious symptoms, and this should be reinforced periodically. However, the survival is poor in the metastatic setting, and the treatment of choice is debatable, some patients may benefit from metastasectomy and local radiotherapy.

## Introduction

Malignant melanoma (MM) is a cancer of the skin, which origins typically from melanocytes. The incidence of MM has been rising on average 1.4% each year over the last ten years, and its estimated new cases and deaths are about 76000 and 10000 people in 2016, respectively [1]. This medical condition usually presents in a highly aggressive fashion with an extreme tendency to metastasis throughout the body most commonly to the lungs, brain, liver, and gastrointestinal tract. In the metastatic setting, median survival declines significantly to 6-7.5 months with a 5-year survival of approximately 6% [1]. The treatment of choice in the metastatic setting of MM is not adequately studied. However, some therapeutic strategies including multi-agent chemotherapy regimens (dacarbazine, platinum analogs, bis-chloronitrosourea [BCNU], vinca alkaloids or taxanes) and/or immunotherapy (interferon α, interleukin 2) had been introduced [2]. We describe a patient with MM that was treated by local radiotherapy and systemic chemotherapy. During follow up, a locally advanced metastasis of paranasal sinuses was detected, and the patient underwent metastasectomy, and local therapy for MM showed complete responses with long-term survival.

## Case Presentation

A 31-year-old man with no past medical history presented with an extensive ulcerative black lesion on the posterior of the right thigh accompanying with swelling of right groin and right side of the neck. He mentioned the protuberancy behind the right thigh from one year ago, which got ulcerated after eight months and simultaneously inguinal and neck mass appeared. Physical examinations revealed a 15×15 cm black lesion with patchy ulceration on the posterior of the right thigh. Also, there were multiple unremarkable lymph nodes in the right inguinal region (their estimated total size was 15×10 cm) and a 1.5 cm lymph node in the submandibular nodes (level Ib). Other places of skin and lymph node region were evaluated, and no specific clinical finding was detected. The general differential diagnosis a cutaneous lesion with described details were atypical mole (Clark nevus or dysplastic nevus), basal cell carcinoma, blue nevi, cherry hemangioma, halo nevus, keloid and hypertrophic scar, keratoacanthoma, lentigo, melanocytic nevi, nevi of Ota and Ito, seborrheic keratosis, Spitz nevus, vitiligo, squamous cell carcinoma, dermatofibroma, and dermatologic manifestations of metastatic carcinomas, which might be accompanied with lymph nodes involvement. Therefore, the patient underwent an incisional biopsy of thigh lesion.

Histopathologic evaluation showed malignant neoplastic proliferation, partly ulcerated surface of the epidermis as patternless sheets of highly atypical pleomorphic epithelial immature cells with polyhedral shape, irregular vesicular nuclei, enlarged nucleoli, and moderate eosinophilic cytoplasm that were mostly compatible with undifferentiated skin carcinoma or amelanotic MM. Immunohistochemistry staining were performed for CK, LCA, CD117, CD34, S100, and Melan-A, which were labeled only for S100 protein and markers of melanocytic differentiation; Melan-A. Morphologic and immunohistochemical findings were consistent with amelanotic MM.

Following the final diagnosis, the patient underwent contracted thoracic, abdominal, and pelvic computed tomography (CT) scan confirming metastatic lymphadenopathy of right inguinal region.

The patient received Dartmouth chemotherapy for eight cycles [2] that include cisplatin 40 mg and dacarbazine (DTIC) 40 mg, both on days 1–3, given in three weekly cycles and BCNU 120 mg on day 1 and 80 mg on day 2 of cycles 1, 3, and 6. Also, tamoxifen 20 mg daily was prescribed continuously. The patient had not previously received DTIC chemotherapy. Dose modification and bone marrow support using granulocyte-colony stimulating factor (GCSF) were applied because of hematologic toxicity. After 5 cycles of chemotherapy, the patient developed grade I neutropenia; therefore, five ampules of GCSF (300 mg) were added to his regimen. However, after this cycle, the patient developed grade I neutropenia again resulting in a 20% dose reduction. Patient evaluation during chemotherapy cycles showed a dramatic response with a significant size reduction of thigh lesion (size of the lesion after 4 chemotherapy cycles was 2 cm) and disappearance of inguinal and cervical lymph nodes. Then after, the patient received local radiotherapy of thigh lesion (36 Gy in 6 fractions) and inguinal and cervical lymph nodes (50 Gy in 25 fractions). This treatment regimen was tolerated very well. Physical examination at the end of radiotherapy revealed no abnormal findings and thigh lesion was healed completely.

**Figure 1:**
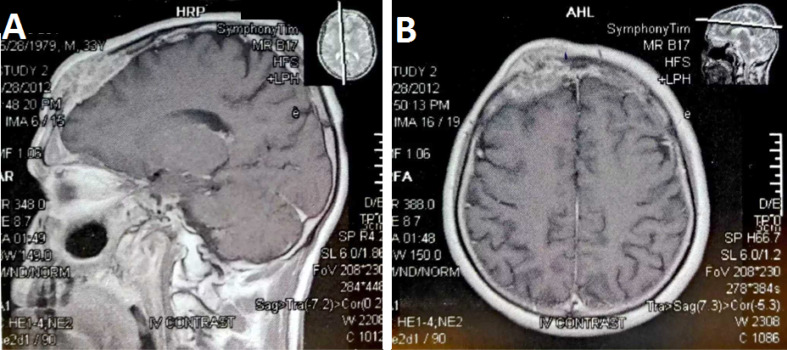
Sagittal (A) and axial (B) CT scan of face revealed an extensive tumoral mass in maxillary, sphenoid, and ethmoid sinuses and, also, a soft tissue lesion in the left frontal sinus with bone destruction on that biopsy confirmed metastatic MM

About 15 months later, the patient presented with nasal congestion and CT scan of face revealed an extensive tumoral mass in maxillary, sphenoid, and ethmoid sinuses and, also, a soft tissue lesion in the left frontal sinus with bone destruction on the that biopsy confirmed metastatic MM (Figure-1). Then, he underwent endonasal endoscopic excision of the mass. During operation, surgeon reported a tumor invasion to anterior cranial fossa, dural and right periorbital region and therefore R2 resection was performed. Because of gross tumor remnant in paranasal sinuses, the patient received radiotherapy of the nasal cavity, paranasal sinuses, and base of the skull (50 Gy in 25 fractions).

The patient was followed-up clinically and serial CT scans or MRI periodically until seven years. Follow-up imaging showed a gradual regression of paranasal sinuses tumor, and he is completely clinical disease-free for approximately seven years.

## Discussion

In this case report, we described a patient with none-metastatic MM, which was admitted in our clinic and underwent systemic chemotherapy (Dartmouth protocol) and local radiotherapy. One year later, the patient presented with extensive tumoral metastases in paranasal sinuses with bone destruction/dural involvement and underwent endonasal endoscopic metastasectomy and radiotherapy of the nasal cavity, paranasal sinuses, and base of the skull. He is followed-up for seven years, and there was no evidence of recurrence (on the thigh or in paranasal sinuses) or new metastasis.

Based on the final version of American Joint Committee on Cancer, the primary tumor thickness, ulceration, lymph node, and distant metastases are used for staging of melanoma [3]. The main cause of death in patients with melanoma is widespread metastases that occur in regional lymph nodes, as in-transit lesions, or in distant organs. The homing of neoplastic melanoma cells are primarily caused by lymph flow and chemotaxis [4].

The median survival of metastatic MM is less than eight months, with a 5-year survival of approximately 6% [1]. Although long-term survival is rare, few reports describe cases after chemotherapy with long-term control [5-7]. In 2005, Durando et al. report that long-term remission of 6,7,9,12, and 14 years after Cystemustine therapy for metastatic MM [8]. Most of these studies are small with a limited number of patients. In a 1994 study by Samuel et al. reported that procarbazine, vincristine, and lomustine chemotherapy regimen is associated by overall survival of 22 weeks; however, two patients remaining in complete remission at 6 and 6.5 years [9]. One hundred sixty-nine patients with metastatic MM were studied in 1996 by Petit et al. and results showed that only five patients (out of 169 patients) had long-term survival seven years after chemotherapy by Fotemustine regimen [10]. The reason why some patients have a very dramatic response and long-term control has not been fully elucidated.

## Conclusion

In our patient, ethmoid and maxillary sinus involvements by MM without visceral metastases have been reported, but for frontal sinus, involvement has not been found. Due to the rarity of this conflict, if the primary site is controlled, in the absence of visceral metastatic for metastatic involvement of the sinus, can be used same as the initial conflict and after the maximum resection surgery, radiotherapy can be used. In this case report also according to R2 surgery, radiotherapy is necessary, and with this therapy, long-term survival without disease for the patient was achieved. MM could be associated with metastasis to any regions and clinicians should be aware of this capability of this tumor and perform complete investigation in the presence of any suspicious symptoms. However, the survival is poor in the metastatic setting, and the treatment of choice is debatable, some patients may benefit from metastasectomy and local radiotherapy. Metastatic MM of the paranasal sinuses is rare and has a poor prognosis.

## Conflict of Interest

The authors have no financial disclosures or multiplicity of interests.
